# Specific Neuropilins Expression in Alveolar Macrophages among Tissue-Specific Macrophages

**DOI:** 10.1371/journal.pone.0147358

**Published:** 2016-02-22

**Authors:** Naing Ye Aung, Rintaro Ohe, Hongxue Meng, Takanobu Kabasawa, Suran Yang, Tomoya Kato, Mitsunori Yamakawa

**Affiliations:** Department of Pathological Diagnostics, Yamagata University Faculty of Medicine, Yamagata, Japan; Institut Pasteur, FRANCE

## Abstract

In the immune system, neuropilins (NRPs), including NRP-1 and NRP-2, are expressed in thymocytes, dendritic cells, regulatory T cells and macrophages. Their functions on immune cells around the neoplastic cells vary into pro-angiogenesis, tumor progression and anti-angiogenesis according to their ligands. Even though NRPs expression on malignant tumors and immune system has studied, a PubMed-based literature query did not yield any articles describing NRPs expression on tissue-specific macrophages. The aims of this study were (i) to detect NRPs expression on tissue-specific macrophages in the brain, liver, spleen, lymph node and lung; (ii) to observe NRPs expression in classes of macrophages, including alveolar macrophages (AMs), bronchial macrophages (BMs), interstitial macrophages (IMs), intravascular macrophages (IVMs) and macrophage subsets (M1, M2 and Mox) in lung; and (iii) to detect the co-expression of NRPs and dendritic cell-specific ICAM-3-grabbing nonintegrin (DC-SIGN) in AMs. Both NRPs were specifically detected in AMs among tissue-specific macrophages by immunohistochemistry (IHC). NRPs mRNA expression levels were characterized in normal lung by reverse transcriptase polymerase chain reaction (RT-PCR) and *in situ-*polymerase chain reaction (*in situ*-PCR). The expression of both NRPs was detected in AMs, BMs and IVMs by IHC. The frequency of NRPs^+^ AMs in lung tissue adjacent to the cancer margin was significantly higher than the frequencies in inflamed and normal lung tissue. Double and triple IHC demonstrated that NRPs are expressed on all macrophage subsets in lung. Double IHC showed co-expression of DC-SIGN and NRPs in AMs. This study demonstrated for the first time the specific expression of both NRPs in AMs among tissue-specific macrophages and their expression on M1, M2 and Mox macrophages. Furthermore, the possible origin of AMs from blood monocytes could be suggested from a co-expression of NRPs and DC-SIGN.

## Introduction

Neuropilins (NRPs) are 120–130 kDa transmembrane non-tyrosine kinase glycoproteins, identified as co-receptors for semaphorin (SEMA) and vascular endothelial growth factor (VEGF). The two neuropilins, neuropilin-1 (NRP-1) and neuropilin-2 (NRP-2), are 44% similar at the amino acid level and consist of a large N-terminal extracellular domain, a short transmembrane domain and a small cytoplasmic domain [1,2]. The extracellular region is divided into three domains. Detection analysis of the domains suggests that the a1/a2 and b1/b2 domains are involved in class 3 SEMA functioning as receptors for neuronal guidance and the b1/b2 is also involved in the binding of VEGF_165_. Presence of the a1/a2 domain, although not essential, also enhances VEGF_165_ binding to NRP-1 [2]. The c- and transmembrane domains are involved in receptor dimerization, a requirement of SEMA 3A signaling, with the c-domain thought to play a role in NRP-1 oligomerization. A neuropilin interacting protein (NIP or synectin) containing cytoplasmic PDZ (PSD-95/Dlg/ZO-1)-domain has also been identified and its domain is responsible for interaction with VEGFR-2 [3]. Both classes of NRPs also control endothelial cell behavior. NRP-1 acts as a VEGF-A isoform receptor in blood vascular endothelium and as a semaphorin receptor in lymphatic valve endothelium, whereas NRP-2 promotes lymphatic vessel growth induced by VEGF-C [3]. NRPs can also exist as soluble isoforms with naturally occurring soluble NRP-1 (sNRP-1) and sNRP-2 functioning as natural inhibitors, where sNRP-1 acts as a competitive antagonist of VEGF_165_. Furthermore, it is also known that NRPs bind to members of the fibroblast growth factor family, along with galectin-1, hepatocyte growth factor/scatter factor, anti-thrombin III, prion protein, transforming growth factor-β and platelet-derived growth factor [4–8].

Expression of both NRPs has been demonstrated in different non-neoplastic epithelial cells, including epithelial cells of the gastrointestinal tract, skin, breast, urinary tract, and respiratory tract. In addition, epithelial tumors that express NRPs include esophageal carcinoma, gastric carcinoma, colorectal carcinoma, lung carcinoma, prostate carcinoma, breast carcinoma and others involved in angiogenesis and tumor progression, as recently reviewed [9]. Gèrald J, *et al*. reported that NRP-1 is expressed by thymocytes, plasmacytoid dendritic cells and regulatory T (Treg) cells in the immune system [10]. According to recent work, NRP-2 expression has been found in cells of the monocyte/macrophage family, including alveolar macrophages [11]. The functions of NRPs on immune cells around the neoplastic cells vary into pro-angiogenesis, tumor progression and anti-angiogenesis according to their ligands. However, their specific function on immune system is still obscure. There are a few of studies on NRPs expression in lung and brain using mouse tissues, however the aims of these studies were focused on angiogenesis, tumor progression and expression on normal tissues. In addition, a PubMed-based literature query for NRPs expression in tissue-specific macrophages did not yield any articles.

Terminal differentiation of blood monocytes into macrophages was driven by two growth factors, macrophage colony-stimulating factor and chemokine (C-XC motif) ligand 4. Macrophages can be divided into M0, M1, M2, M4, Mox, HA-Mac, M(Hb) and Mhem [12]. Nevertheless, M1, M2 and Mox can be investigated by immunohistochemistry, in human.

The origins of tissue-specific macrophages differ from one another. Under conditions other than steady state, alveolar macrophages originate from blood monocytes. Tissue-specific macrophages from brain, liver and spleen are thought to have differentiated from local proliferating precursors [13]. Differentiation of macrophages may also be from blood monocytes and, blood monocytes and monocyte-derived dendritic cells express NRP-1 and DC-SIGN (dendritic cell-specific ICAM-3-grabbing nonintegrin). It can be suggested that macrophages differentiation from blood monocytes and transmigration through the vascular endothelia may closely related with NRPs and DC-SIGN. However, no studies to date have described the co-expression of and the correlation between NRPs and DC-SIGN on alveolar macrophages.

The aims of this study were (i) to detect the expression of NRPs on tissue-specific macrophages in the brain, liver, spleen, lymph node and lung; (ii) to observe NRPs expression in classes of macrophages, including alveolar macrophages (AMs), bronchial macrophages (BMs), interstitial macrophages (IMs), intravascular macrophages (IVMs) and macrophage subsets (M1, M2 and Mox) in lung; and (iii) to detect the co-expression of NRPs and DC-SIGN in AMs.

## Materials and Methods

### Tissue specimens

To explore NRPs expression in tissue-specific macrophages, tissue specimens (n = 5) of brain, liver and lung tissues were obtained from remote to the cancer nest, whereas spleen and lymph node tissues (n = 5) were from the patients with reactive hyperplasia. To observe NRPs expression in classes and subsets of macrophages in lung, pulmonary tissues, including lung cancer tissue, tissue remote to the cancer nest (physiologically normal lung) and inflamed lung tissue were obtained from Yamagata University Hospital between 2009 and 2013 (Table 1). All of the specimens were obtained under written consent from each patient. This study was approved by the Research Ethics Committee (H25-117) of Yamagata University Faculty of Medicine, Yamagata, Japan.

**Table 1:** Clinical characteristics of cases used in this study.

Cases/Diseases	Value
Lung tissue (remote to cancer) (n = 5)	
Male/Female	2/3
Mean age, years (range)	66.2 (61–72)
Inflamed lung (n = 20)	
Interstitial pneumonia (n = 5)	
Male/Female	3/2
Mean age, years (range)	59.6 (41–71)
Organizing pneumonia (n = 4)	
Male/Female	4/0
Mean age, years (range)	55.7 (48–75)
Bronchopneumonia (n = 4)	
Male/Female	2/2
Mean age, years (range)	76.0 (56–85)
Lobar pneumonia (n = 1)	
Male/Female	0/1
Mean age, years (range)	74.0 (-)
Epithelioid granuloma (n = 6)	
Male/Female	2/4
Mean age, years (range)	54.5 (20–78)
Lung cancer (n = 33)	33
Adenocarcinoma (n = 15)	
Male/Female	8/7
Mean age, years (range)	68.6 (43–77)
Smoking habits (yes/no/unknown)	(8/3/4)
Squamous cell carcinoma (n = 15)	
Male/Female	12/3
Mean age, years (range)	72.4 (56–84)
Smoking habits (yes/no/unknown)	(11/3/1)
Small cell carcinoma (n = 3)	
Male/Female	2/1
Mean age, years (range)	75.3 (66–85)
Smoking habits (yes/no/unknown)	(0/1/2)

Tissues were fixed in buffered 10% formalin for 6–12 hours at room temperature, embedded in paraffin and used for single immunohistochemistry (IHC), double IHC, triple IHC, double immunofluorescence (IF), single IHC after single IF, reverse transcriptase polymerase chain reaction (RT-PCR) and *in situ*-polymerase chain reaction (*in situ*-PCR).

### Single immunohistochemistry (Single IHC)

IHC was performed using antibodies against NRP-1 (rabbit polyclonal, Abcam, Cambridge, United Kingdom), NRP-1 (A-12, mouse IgG1, Santa Cruz Biotechnology, CA, USA), NRP-1 (rabbit polyclonal, Invitrogen, Frederick, MD, USA), NRP-2 (goat polyclonal, R&D Systems, Minneapolis, MN, USA), CD68 (PG-M1, mouse IgG1κ, DAKO, Glostrup, Denmark), CD163 (10D6, mouse IgG1, NOVOCASTRA, Newcastle, United Kingdom), HO-1 (D-8, mouse IgG1, Santa Cruz Biotechnology) and DC-SIGN (rabbit polyclonal, IgG, Abcam). We used arterial, venous and lymphatic endothelia as positive controls for NRP-1 and NRP-2 [14]. Phosphate-buffered saline (PBS, 0.01 M, pH 7.4), Universal Negative Control-Mouse (N1698; DAKO) and Universal Negative Control-Rabbit (N1699; DAKO) were used as negative controls.

Three-micrometer-thick cut sections were deparaffinized. Endogenous peroxidase activity was blocked with methanol containing 0.3% hydrogen peroxide for 30 min on ice. Antigen retrieval was performed using EDTA (Antigen Retrieval Solution pH 9; Nichirei Biosciences, Tokyo, Japan) or citric acid (Antigen Retrieval Solution pH 6; IATRON LABORATORIES INC., Tokyo, Japan) in an autoclave (2 atmospheres, 121°C, 20 min) and 7 min for 3 times in a microwave. Sections were incubated with primary antibodies at room temperature overnight. The labeled streptavidin-biotin peroxidase method (UltraTech HRP Streptavidin-Biotin Detection system, PN IM2391; Immunotech, Marseille, France), Histofine SAB-PO (anti-goat) kit (Nichirei Biosciences, Tokyo, Japan) and EnVision+ System-HRP labeled polymer (anti-rabbit, DAKO, Carpinteria, CA, USA) were used. Positive reactions were detected as a brown coloration with 3,3’-diaminobenzidine tetrahydrochloride (DAB) (Dojindo, Kumamoto, Japan). Sections were then counterstained with hematoxylin. The expression levels of NRP-1 and NRP-2 in tissue-specific macrophages in brain, lung, liver, spleen and lymph node were confirmed with IHC on serial sections, including an antibody cocktail solution for CD68 and CD163.

Positive cells, as determined by IHC for NRP-1, NRP-2 and DC-SIGN, were counted as AMs in the alveolar space, BMs in the bronchial lumen or endobronchial area, IMs in the peribronchial area or interstitium and IVMs in the vascular lumen. NRP-1- and NRP-2-positive macrophages in alveolar space adjacent to lung cancer, in inflammatory alveolar space in inflamed lung and in alveolar space in lung tissue remote to the cancer nest were counted. Positive cells were counted in 10 areas in a high-power view field (HPF) by two experienced observers in a blinded manner.

### Double immunohistochemistry (Double IHC)

Positive reaction of the first antibody was detected as a dark blue coloration with the 5-bromo-4-chloro-3-indolyl-phosphate/nitro blue tetrazolium (BCIP/NBT) substrate system (DAKO), and positive reaction of the second antibody was detected as a dark red coloration with AEC (3-amino-9-ethylcarbazole) (Nichirei Biosciences, Tokyo, Japan). The paired antibodies used for double IHC were NRP-1/CD68, NRP-1/CD163, NRP-1/HO-1, NRP-1/DC-SIGN, NRP-2/CD68, NRP-2/CD163, NRP-2/HO-1 and CD68/CD163. Percentages of CD68^+^CD163^-^ AMs in lung tissue adjacent to the cancer margin were calculated from the summation of CD68^+^CD163^-^ AMs and the summation of double-positive and CD68^-^CD163^+^ AMs in the same areas in 10 HPFs for 5 cases of lung cancer. For the correlation between NRP-1 and DC-SIGN, positive cells were counted in the same areas in 10 HPFs for 5 cases of lung cancer. Counterstaining was not performed.

### Triple immunohistochemistry (Triple IHC)

Triple IHC was performed to detect CD68, CD163 and NRP-1 expression in the same AMs in lung tissue adjacent to the cancer margin. Positive reaction of the first antibody (CD68) was detected as a brown coloration with DAB, and positive reaction of the second antibody (CD163) was detected with a pink or light red coloration with the New Fuchsin Substrate System (DAKO). The True Blue^™^ Peroxidase Substrate System (KPL, Gaithersburg, MD, USA) was used for color detection of the third antibody (NRP-1). Counterstaining was not performed.

### Double immunofluorescence (Double IF)

Immunofluorescence double staining using paraffin-embedded tissue sections was performed as previously described [15]. IF was performed using antibodies against NRP-1 (rabbit polyclonal, Abcam, Cambridge, United Kingdom), NRP-2 (C-9, mouse monoclonal IgG_2b_, Santa Cruz Biotechnology, CA, USA), CD68 (PG-M1, mouse IgG1κ, DAKO, Glostrup, Denmark), CD163 (10D6, mouse IgG1, NOVOCASTRA, Newcastle, United Kingdom), HO-1 (D-8, mouse IgG1, Santa Cruz Biotechnology) and DC-SIGN (rabbit polyclonal, IgG, Abcam). In brief, following antigen retrieval, tissue sections were incubated overnight with a cocktail of primary antibodies, followed by fluorescein-conjugated AffiniPure goat anti-rabbit IgG (H+L) (Jackson ImmunoResearch Laboratories, West Grove, PA) and rhodamine-conjugated AffiniPure donkey anti-mouse IgG (H+L) (Jackson ImmunoResearch Laboratories). Double IF was done to explore the co-expression of CD68, CD163 and HO-1 on NRP-1^+^ AMs, and co-expression of DC-SIGN on NRP-2^+^ AMs.

### Single IHC after single IF

For the same host species of primary antibodies, single IHC after single IF were done. After observations of first primary antibodies of same host species by single IF under fluorescence microscopy, single IHC was done for second primary antibodies of same host species. The pictures were taken at same cells on same slides. Single IHC after single IF were performed to study co-expression of CD68, CD163 and HO-1 on NRP-2^+^ AMs.

### Reverse transcriptase polymerase chain reaction (RT-PCR)

Formalin-fixed and paraffin-embedded tissues from the brain (n = 2), liver (n = 2), spleen (n = 2), lymph node (n = 2) and lung (n = 3) were used for mRNA detection by RT-PCR. mRNA was extracted with a WaxFree RNA^™^ RNA Extraction Kit (TrimGen, Sparks, MD, USA) according to the manufacturer’s protocol. mRNA (< 5 μg) was used as a template for the synthesis of cDNA (20 μl) with a PrimeScript^™^ II 1^st^ strand cDNA Synthesis Kit (TAKARA BIOTECHNOLOGY, Shiga, JAPAN). Three microliters of cDNA was PCR-amplified utilizing EmeraldAmp^®^PCR Master Mix (TAKARA) and specific primers. The primer sequences were 5’-TGAGCCCTGTGGTTTATTCC-3’ and 5’-CGTACTCCTCTGGCTTCTGG-3’ for human NRP-1 (120 bp), 5’-GTGGTTCATCTTGACCTTGT-3’ and 5’-ATTCTTCTTCTGCAACCTCA-3’ for human Nrp-2 (257 bp) [16,17] and 5’-GCACCGTCAAGGCTGAGAAC-3’ and 5’-TGGTGAAGACGCCAGTGGA-3’ for human glyceraldehyde-3-phosphate dehydrogenase (GAPDH; 137 bp) as the internal control. cDNA was amplified as follows: 94°C for 10 min; 40 cycles of 94°C for 30 sec, 57°C for 45 sec and 72°C for 30 sec; and 1 cycle of 72°C for 6 min in a Veriti^™^ 96 Well Thermal Cycler (Applied Biosystems, Foster city, CA, USA). Final PCR products were placed at 4°C until electrophoresis. We used primers for human NRP-1 designed by PrimerBank. The PCR products were electrophoresed in a 4% agarose gel and stained with ethidium bromide.

### *In situ*-polymerase chain reaction (*in situ*-PCR)

We examined lung tissue remote to the cancer nest to detect the mRNA expression levels of NRP-1 and NRP-2 in AMs. The conditions used for *in situ*-PCR were as described elsewhere [18,19]. Briefly, dewaxed and rehydrated 3–5 μm paraffin sections were fixed in 4% paraformaldehyde for 4 hours at room temperature. After fixation, the slides were incubated in pepsin (8 mg/ml) for 75 min at 37°C. After protein digestion, the slides were air-dried and incubated in DNase digestion solution (DNase I recombinant, RNase-free, Roche Diagnostics, Mannheim, Germany) at 37°C overnight.

After DNase digestion, a reverse transcriptase reaction was performed in the solution provided in the PrimeScript II cDNA synthesis kit (TAKARA) according to the manufacturer’s protocol. Coverslips were placed over the solutions in the DNase digestion and reverse transcriptase reactions to prevent evaporation of the solutions. Slide sealers (TAKARA) were placed on the slides around the tissue prior to PCR.

The *in situ*-PCR reaction mix consisted of PCR buffer with 15 mM MgCl_2_, PCR digoxigenin (DIG) labeling mix, Taq DNA polymerase and primer pairs for each NRP-1 and NRP-2 (same primer pairs as RT-PCR). A total of 125 μl of PCR reaction mix was applied to the tissue sections, which were then sealed with slide sealers. Slides were placed into a MasterCycler^®^ PCR thermal cycler (Eppendorf, Hamburg, Germany) for 1 cycle of 94°C for 10 min; 30 cycles of 94°C for 30 sec, 57°C for 45 sec and 72°C for 30 sec; and 1 cycle of 72°C for 6 min. The location of DIG incorporated into PCR amplicons was detected by an alkaline phosphatase assay using anti-DIG-alkaline phosphatase Fab fragments (1:100 dilution) (Roche Diagnostics), which were incubated with the samples for 30 min at 37°C. The *in situ*-PCR product was detected using a Vulcan fast red chromogen kit 2 (BIOCARE MEDICAL, Concord, CA, USA) and was counterstained with hematoxylin. Tissue sections without primers and without reverse transcriptase reactions were used as negative controls.

### Statistical analysis

The Kruskal-Wallis test was performed for comparisons of IHC-positive cells and the different groups. The Mann-Whitney *U*-test was performed for the comparison of positive cells for each of the 2 groups. Data were analyzed using SPSS (Statistical Package for the Social Sciences) ver.13.0 software (SPSS Inc, Chicago, IL, USA) with the level of significance at *p*< 0.05. The correlation between NRP-1 and DC-SIGN was quantified using the Spearman rank correlation method and the Pearson correlation method.

## Results

### Expression of NRP-1 and NRP-2 on tissue-specific macrophages in the brain, lung, liver, spleen and lymph node

**Fig 1:**
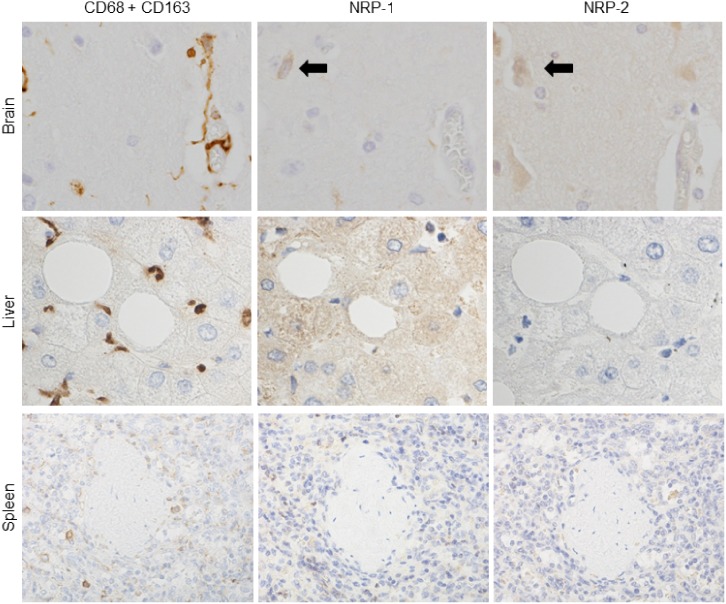
NRPs expression in tissue-specific macrophages compared to immunostaining with a cocktail of anti-CD68 and anti-CD163 antibodies. Tissue-specific macrophages were recognized by immunostaining with a cocktail of anti-CD68 and CD163 antibodies in serial sections. NRP-1 and NRP-2 expression was not observed in tissue-specific macrophages of brain (microglia), liver (Kupffer cells) and spleen (red pulp macrophages). Black arrows indicate the neuronal staining of NRPs in brain. Serial sections were counterstained with hematoxylin. NRP-1, neuropilin 1; NRP-2, neuropilin 2.

**Fig 2:**
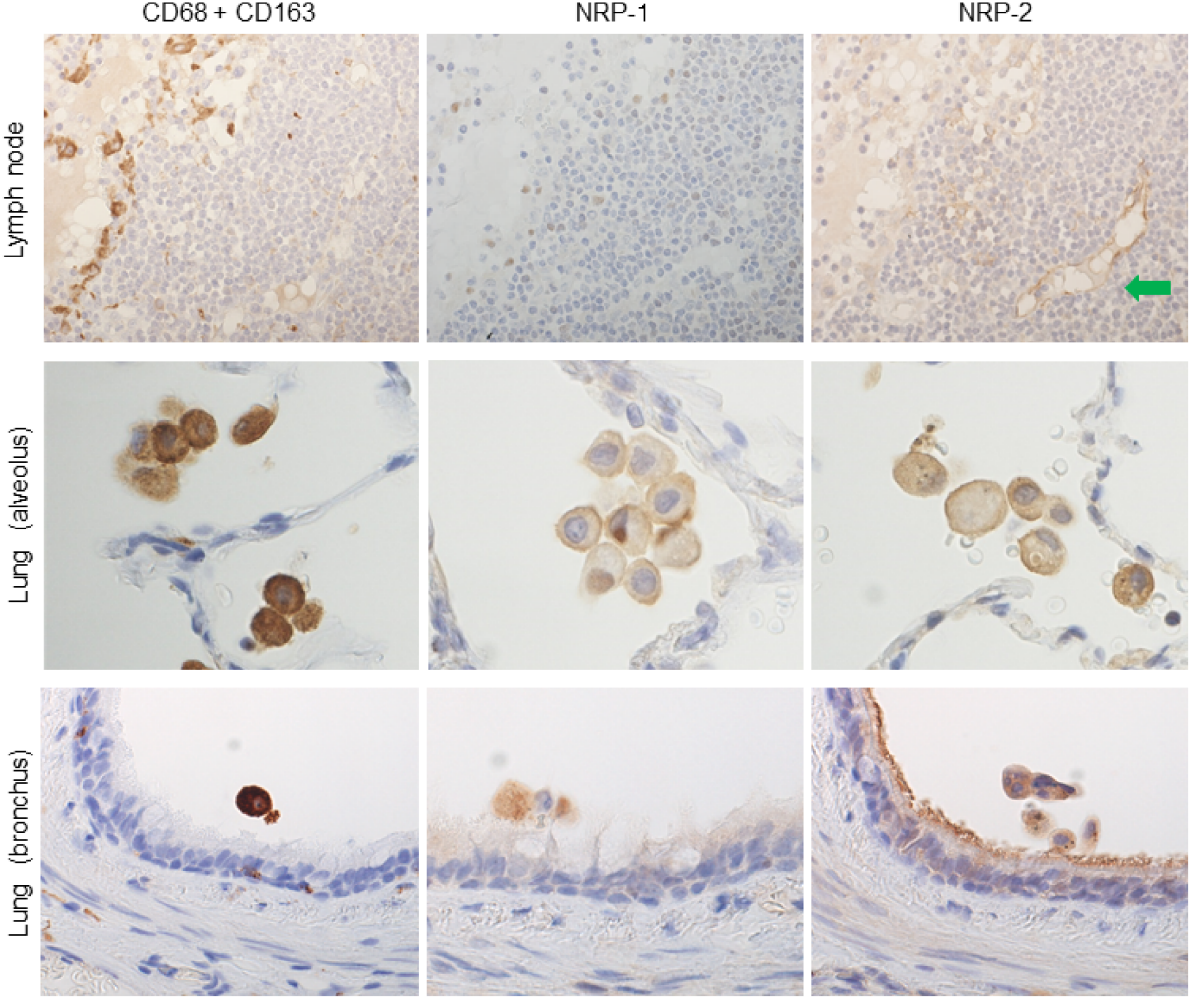
NRPs expression in tissue-specific macrophages compared to immunostaining with a cocktail of anti-CD68 and anti-CD163 antibodies. Tissue-specific macrophages were recognized by immunostaining with a cocktail of anti-CD68 and CD163 antibodies in serial sections. NRP-1 and NRP-2 expression was detected in alveolar macrophages in lung, but not in lymph node (sinus macrophages). And NRP-1 and NRP-2 also expressed on bronchial macrophages. Green arrow indicates NRP-2 expression on lymphatic vascular endothelium, used as positive control. Serial sections were counterstained with hematoxylin. NRP-1, neuropilin 1; NRP-2, neuropilin 2.

To investigate NRP-1 and NRP-2 expression in tissue-specific macrophages, single IHC for NRP-1 and NRP-2 and immunostaining with cocktail antibodies for CD68 and CD163 in serial tissue sections were performed. NRP-1 and NRP-2 were specifically expressed in AMs among tissue-specific macrophages (microglia in the brain, Kupffer cells in the liver, red pulp macrophages in the spleen and sinus histiocytes in the lymph node) (Figs 1 and 2). In addition, NRP-1 and NRP-2 expression was detected on neurons in brain tissue (Fig 1). In lung tissue, NRP-1 expression was observed in the nuclei and cytoplasm of neutrophils, some reactive lymphocytes, plasma cell-like lymphocytes and respiratory epithelial and glandular epithelial cells. NRP-2 expression in lung tissue was observed in respiratory epithelial and glandular epithelial cells. Weak NRP-1 expression was observed in the cytoplasm of hepatocytes in liver tissue. In spleen and lymph node, NRP-1 expression was observed in some lymphocytes in the marginal and mantle zones of lymphoid follicles, plasma cell-like lymphocytes and some dendritic cell-like cells. NRP-1 and NRP-2 expression was observed on lymphatic and vascular endothelial cells in brain, lung, liver, spleen and lymph nodes.

**Fig 3:**
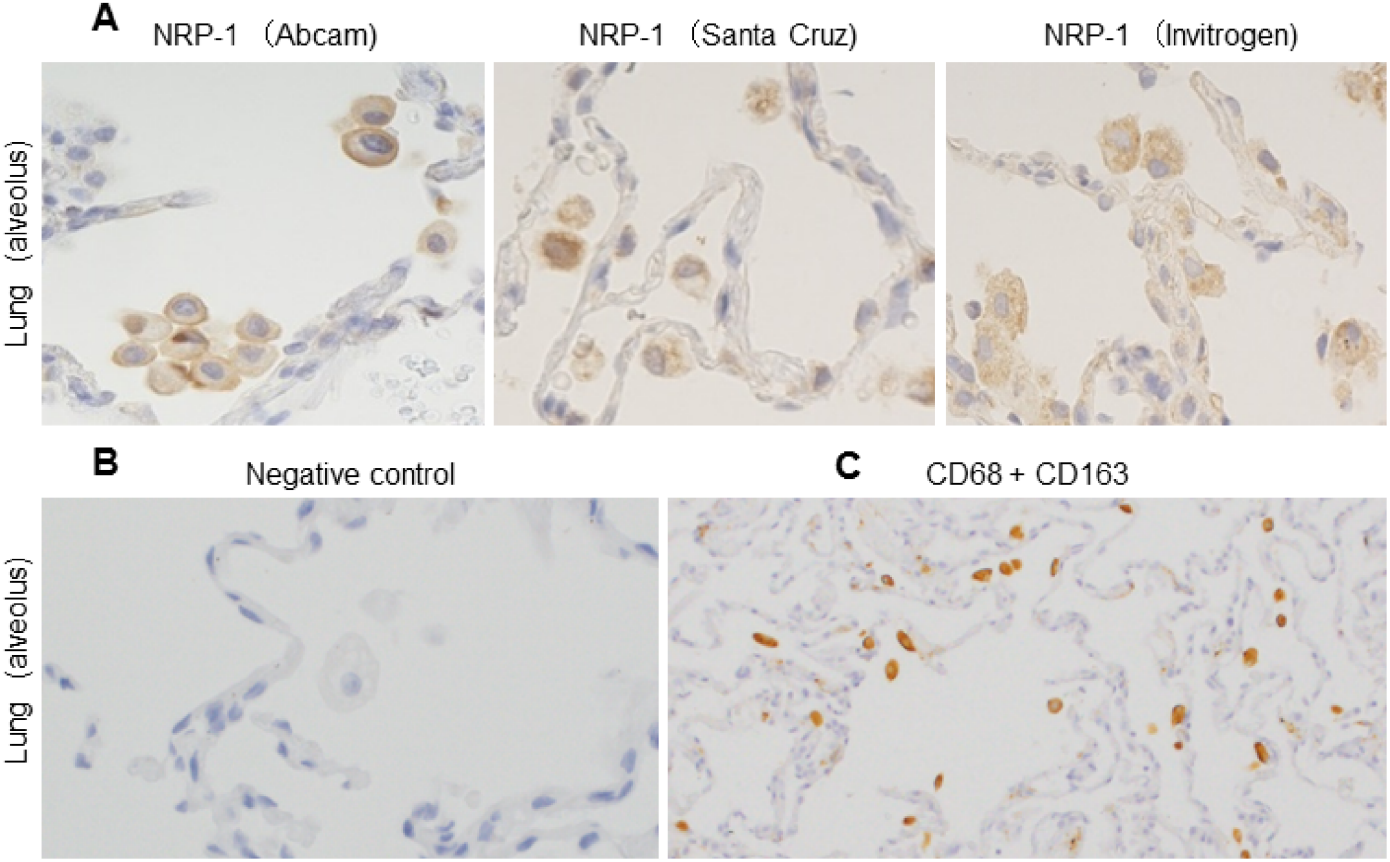
NRPs expression from different sources in alveolar macrophages and, IHC with a cocktail of anti-CD68 and anti-CD163 antibodies in serial section of *in-situ* PCR. (A) Immunohistochemistry showed alveolar macrophages expressing NRP-1 as detected by using 3 different antibodies from Abcam, Santa Cruz Biotechnology and Invitrogen. Serial sections were counterstained with hematoxylin. (B) Isotype or negative control showed no reactivity. It was counterstained with hematoxylin. (C) All alveolar macrophages showed positivity with CD68 and CD163. NRP-1, neuropilin 1.

NRP-1 antibodies from 3 different sources (Abcam, Santa Cruz Biotechnology, and Invitrogen) were tested on the above tissues, and all antibodies showed positive reactions with alveolar macrophages among tissue-specific macrophages (Fig 3). However, the nuclei and cytoplasm of lymphocytes in the marginal and mantle zones of lymphoid follicles, plasma cell-like lymphocytes and some dendritic cell-like cells in spleen and lymph node showed positive reactions only with the NRP-1 antibody from Abcam. Further IHC in this study was performed using the NRP-1 antibody from Abcam.

### NRP-1 and NRP-2 mRNAs expression in the brain, liver, spleen, lymph node and lung by RT-PCR and, NRP-1 and NRP-2 mRNA expression in AMs in the lung by *in situ*-PCR

**Fig 4:**
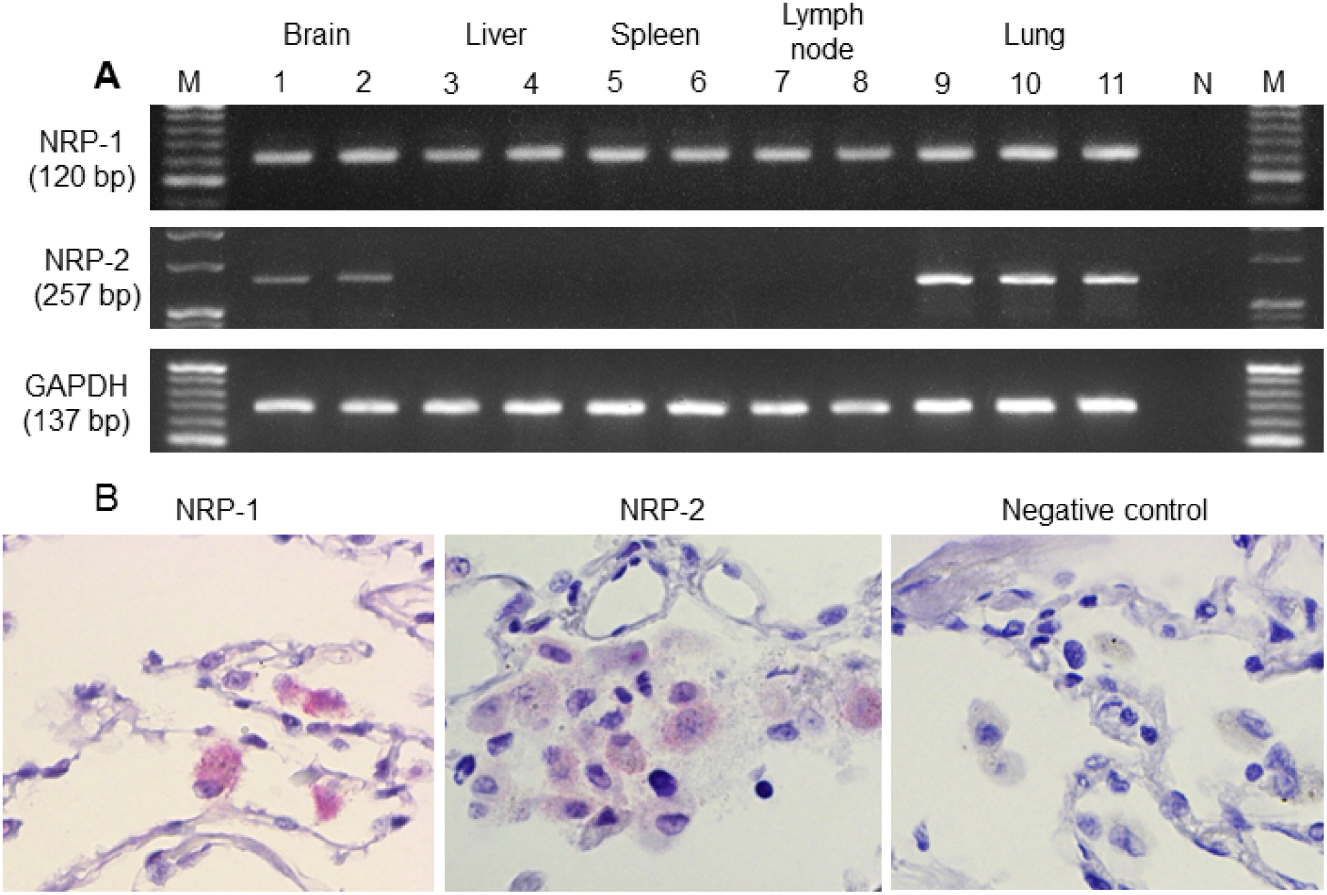
NRPs mRNAs expression in normal tissues (RT-PCR) and on alveolar macrophages in physiologically normal lung (*in situ*-PCR). (A) By reverse transcriptase polymerase chain reaction (RT-PCR), NRP-1 mRNA was expressed in normal brain (*lanes 1*, *2*), liver (*lanes 3*, *4*), spleen (*lanes 5*, *6*), lymph node (*lanes 7*, *8*) and lung (*lanes 9*, *10 and 11*). NRP-2 mRNA was expressed in normal lung and brain but was not expressed in liver, spleen and lymph node. N represents the negative control, and M represents the 20 base-pair DNA ladder. (B) NRP-1 and NRP-2 mRNAs of alveolar macrophages in physiologically normal lung (remote to the cancer nest), as observed by *in situ*-polymerase chain reaction (*in situ*-PCR). NRP-1, neuropilin 1; NRP-2, neuropilin 2; GAPDH, glyceraldehyde-3-phosphate dehydrogenase.

RT-PCR was performed to detect the presence of NRP-1 and NRP-2 mRNAs in the brain, liver, spleen, lymph node and lung. GAPDH mRNA (a housekeeping gene) was expressed in all cases. NRP-1 and NRP-2 mRNAs were expressed in all normal lung tissues. In addition, NRP-1 mRNA was detected in normal brain, liver, spleen and lymph node. However, NRP-2 mRNA was detected only in the normal brain in addition to normal lung (Fig 4A).

To confirm the NRP-1 and NRP-2 mRNA expression levels in AMs, *in situ*-PCR was performed. By *in situ*-PCR, NRP-1 and NRP-2 mRNAs were observed in AMs in lung tissue remote to the cancer nest by coloration with Vulcan fast red stain (Fig 4B). The factor that these NRP^+^ cells are macrophages was confirmed by single immunohistochemistry for antibody cocktail solution of CD68 and CD163 in serial tissue sections. All alveolar macrophages are positive for CD68 and CD163 (Fig 3).

### NRP-1 and NRP-2 expression in classes of lung macrophages in physiologically normal lung

**Fig 5:**
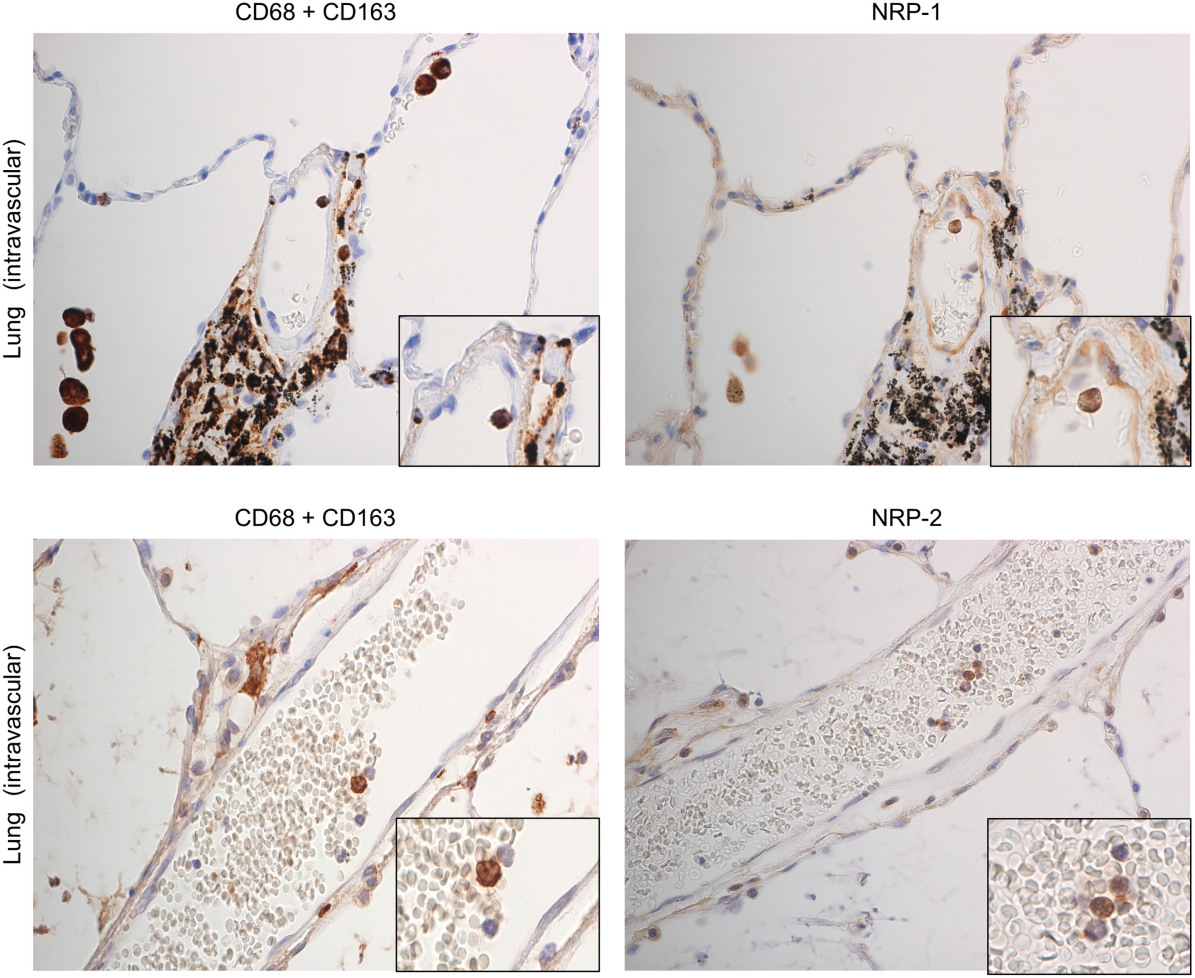
NRPs expression in intravascular macrophages in physiologically normal lung by immunostaining. Intravascular macrophages (blood monocytes) were recognized by immunostaining with a cocktail of anti-CD68 and CD163 antibodies. NRP-1 and NRP-2 expression was observed in intravascular macrophages. Green arrow indicates NRP-1 expression on vascular endothelium, used as positive control. Serial sections were counterstained with hematoxylin. NRP-1, neuropilin 1; NRP-2, neuropilin 2.

To observe NRP-1 and NRP-2 expression in lung macrophages in physiologically normal lung, single IHC for NRP-1 and NRP-2, and IHC for an antibody cocktail solution of CD68 and CD163 in serial tissue sections were performed. Almost all AMs expressed NRP-1 and NRP-2. In addition, NRP-1 and NRP-2 were expressed in BMs (Fig 1) and IVMs (Fig 5). However, NRP-1 and NRP-2 expression was not detected in IMs (data not provided). The frequency of NRP-1 and NRP-2 expression in AMs was significantly higher than that observed in IMs (Table 2). The numbers of NRP-1^+^ and NRP-2^+^ BMs and IVMs were not included in this comparison because the counted areas containing positive cells were not sufficient to account for 10 areas.

**Table 2:** Comparison of neuropilin-1 (NRP-1) and neuropilin-2 (NRP-2) expression in alveolar macrophages and interstitial macrophages in lung tissue remote to the cancer nest (physiologically normal lung) (n = 5).

Type of macrophages	Number of NRP-1 positive cells^A)^ (mean ± SD)	Number of NRP-2 positive cells^A)^ (mean ± SD)
Alveolar macrophages	9.2 ± 3.8*	8.9 ± 3.9*
Interstitial macrophages	0 ± 0*	0 ± 0*

* *p*< 0.01 significant between alveolar macrophages and interstitial macrophages by the Mann-Whitney *U*-test.

^A)^, average number of positive cells/high-power view fields.

### Comparison of NRP-1 and NRP-2 expression in AMs in lung tissue adjacent to the cancer margin, inflamed lung and lung tissue remote to the cancer nest

**Fig 6:**
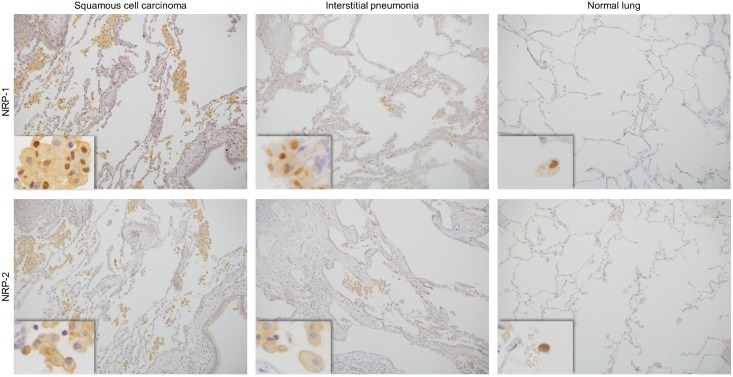
Immunohistochemistry of NRPs expression on alveolar macrophages in lung tissue (adjacent to the cancer margin, inflamed and physiologically normal). NRP-1 and NRP-2 expressed on alveolar macrophages in lung tissue adjacent to cancer (squamous cell carcinoma), inflamed lung (interstitial pneumonia) and physiologically normal lung (remote to the cancer nest). NRP-1, neuropilin 1; NRP-2, neuropilin 2.

To observe the numbers of NRP-1- and NRP-2-positive AMs in different lung conditions, IHC was performed for NRP-1 and NRP-2 in lung tissue adjacent to the cancer margin, tissue remote to the cancer nest (physiologically normal lung) and inflammatory lung tissue. NRP-1 and NRP-2 were expressed in AMs in lung cancer patients both with and without smoking habits. The frequency of NRP-1^+^ AMs in lung tissue adjacent to the cancer margin was significantly higher than the frequency of NRP-1^+^ AMs in inflamed lung and lung tissue remote to the cancer nest. In inflamed lung, the number of NRP-1^+^ AMs was higher than that of NRP-1^+^ AMs in lung tissue remote to the cancer nest. The frequencies of NRP-2^+^ AMs among these lung tissue types were the same as those for NRP-1^+^ AMs (Fig 6, Table 3). NRP-1 and NRP-2 expression was also observed in some lung cancer cells (data not provided).

**Table 3:** Comparison of neuropilin-1 (NRP-1) and neuropilin-2 (NRP-2) expression on alveolar macrophages in lung cancer adjacent to the cancer margin, lung inflammation and lung tissue remote to the cancer nest (physiologically normal lung).

Cases/Diseases	Number of NRP-1 positive cells^A)^ (mean ± SD)	Number of NRP-2 positive cells^A)^ (mean ± SD)
Adenocarcinoma (n = 15)	38.3 ± 8.9^‡^^,^ ^#^	37.8 ± 9.20^‡^^,^ ^#^
Squamous cell carcinoma (n = 15)	46.7 ± 9.2*^,^ ***	48.1 ± 10.7*^,^ ***
Inflamed lung (n = 20)	25.1 ± 9.1*^,^ **^,^ ^‡^	24.5 ± 12.1*^,^ **^,^ ^‡^
Physiologically normal lung (n = 5)	9.2 ± 3.8**^,^ ***^,^ ^#^	8.9 ± 3.9**^,^ ***^,^ ^#^

* *p*< 0.01 significant between squamous cell carcinoma and inflamed lung;

** *p*< 0.01 significant between inflamed lung and physiologically normal lung;

*** *p*< 0.01 significant between squamous cell carcinoma and physiologically normal lung;

^‡^ *p*< 0.05 significant between adenocarcinoma and inflamed lung and

^#^ *p*< 0.01 significant between adenocarcinoma and physiologically normal lung by the Mann-Whitney *U*-test.

^A)^, average number of positive cells/high-power view fields.

The frequencies of NRP-1- and NRP-2-positive alveolar macrophages adjacent to small cell carcinomas (n = 3) were also significantly higher than the frequencies of NRP-1- and NRP-2-positive alveolar macrophages in inflamed lung and lung tissue remote to the cancer nest.

### NRP-1 and NRP-2 expression in macrophage subsets of AMs in lung tissue adjacent to the cancer margin

**Fig 7:**
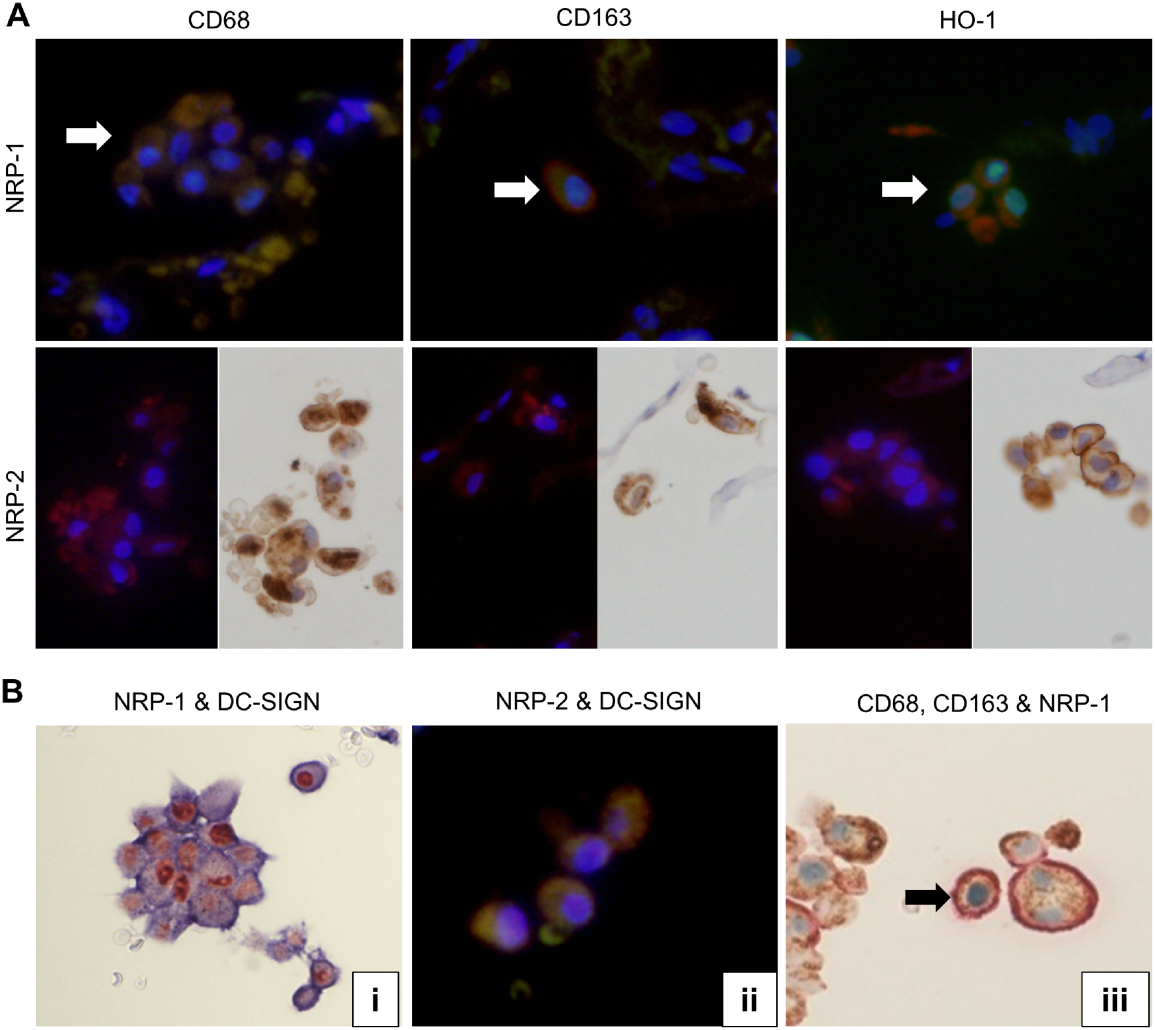
Double immunostaining (NRPs vs CD68, CD163, HO-1 and DC-SIGN) and triple immunohistochemistry (CD68, CD163 and NRP-1) of lung tissue adjacent to the cancer. (A) Double IF showed expression of CD68, CD163 and HO-1 (Rhodamine, anti-mouse, red color) on NRP-1^+^(Fluorescein, anti-rabbit, green color) alveolar macrophages. White arrows showed double positive cells. Single IHC after single IF showed NRP-2+ (Rhodamine, anti-mouse, red color) alveolar macrophages also express CD68, CD163 and HO-1 (LSAB, anti-mouse, brown color). (B) (i) Double IHC showed NRP-1(Red) and DC-SIGN (blue) positive alveolar macrophages. (ii) Double IF showed the co-expression of NRP-2 (Rhodamine, anti-mouse, red color) and DC-SIGN (Fluorescein, anti-rabbit, green color) on AMs. (iii) Triple IHC of CD68 (brown), CD163 (light red) and NRP-1 (light blue) showed triple-positive cells (CD68^+^CD163^+^NRP-1^+^; indicated by *black arrow*) and double-positive cells (CD68^+^NRP-1^+^, CD68^+^CD163^+^ and CD163^+^NRP-1^+^). NRP-1, neuropilin 1; NRP-2, neuropilin 2; DC-SIGN, dendritic cell-specific ICAM-3-grabbing nonintegrin.

To detect NRP-1 and NRP-2 expression in macrophage subsets in lung tissue adjacent to the cancer margin, double IHC, double IF and single IHC after single IF were performed. They revealed CD68, CD163 and HO-1 expression in both NRP-1^+^ and NRP-2^+^ AMs (Fig 7A). Double IHC of CD68 and CD163 showed that 20.1% of CD68^+^CD163^-^ AMs were observed among CD68^-^CD163^+^ AMs and double-positive AMs.

Since macrophage subtypes are differentiated into M1 macrophages (identified as CD68^+^ CD163^-^ HO-1^-^ macrophages), M2 macrophages (CD68^+^ CD163^+^ HO-1^-^) and Mox macrophages (CD68^+^ CD163^+^ HO-1^+^), triple IHC was performed in lung tissue adjacent to the cancer margin to observe NRP-1 expression in single CD68^+^ AMs. Triple IHC revealed that NRP-1 was also expressed in CD68^+^CD163^-^ (M1), CD68^-^CD163^+^ (M2/Mox) and CD68^+^CD163^+^ (M2/Mox) AMs (Fig 7B).

### Expression of and correlation between DC-SIGN and NRP-1 in AMs in lung tissue adjacent to the cancer margin

To observe the co-expression of DC-SIGN and NRP-1 in AMs, single and double IHC assays of NRP-1 and DC-SIGN were performed. DC-SIGN expression was also observed on AMs in lung tissue adjacent to the cancer margin. The number of DC-SIGN^+^ AMs in lung tissue adjacent to the cancer margin was significantly higher than the number of DC-SIGN^+^ AMs in lung tissue remote to the cancer nest (Table 4, *p*< 0.05). Double IHC indicated that 95.8% of DC-SIGN^+^ AMs co-expressed NRP-1 and 94.9% for NRP-2, in lung tissue adjacent to the cancer margin (Fig 7B). The correlation between DC-SIGN and NRP-1 expression was quantified using the Spearman correlation method (R = 0.956, *p*< 0.01) and the Pearson correlation method (R = 0.919, *p*< 0.01). The co-localization of NRP-2 and DC-SIGN on AMs was also explored by double IF (Fig 7B). The correlation between DC-SIGN and NRP-2 expression was also quantified using the Spearman correlation method (R = 0.948, *p*< 0.01) and the Pearson correlation method (R = 0.915, *p*< 0.01).

**Table 4:** Comparison of dendritic cell-specific ICAM-3-grabbing nonintegrin (DC-SIGN) expression on alveolar macrophages in lung tissue adjacent to the cancer margin and lung tissue remote to the cancer nest.

Cases/Diseases	Number of DC-SIGN-positive cells^A)^ (mean ± SD)
Lung tissue adjacent to cancer (n = 5)	32.3 ± 12.9*
Lung tissue remote to cancer (n = 5)	8.1 ± 3.1*

* *p*< 0.01 significant between lung tissue adjacent to cancer and lung tissue remote to cancer by the Mann-Whitney *U*-test.

^A)^, average number of positive cells/high-power view fields.

## Discussion

In this study, NRP-1 and NRP-2 expression was examined on tissue-specific macrophages in the brain, liver, spleen, lymph node and lung by IHC. NRP-1 and NRP-2 expression was observed only in AMs among tissue-specific macrophages. mRNAs of NRP-1 and NRP-2 were detected in normal lung and brain by RT-PCR. Furthermore, the detection of NRP-1 mRNA in spleen and lymph node may be due to the presence of NRP-1 expression on lymphocytes, dendritic cells and plasma cell-like lymphocytes in the spleen and lymph node. The detection of NRP-1 mRNA in liver may be explained by the weak positivity observed in normal hepatocytes by IHC. NRP-1 and NRP-2 mRNAs were also detected in neurons the brain by IHC. Fujita H and co-workers investigated the presence of NRP-1 and NRP-2 by PCR, using rat brain [20]. However, there were no detectable amounts of NRP-2 mRNA in the liver, spleen and lymph node. The presence of NRP-1 and NRP-2 mRNAs in AMs was confirmed by *in situ*-PCR. Based on these data, NRP-1 and NRP-2 expression may differ in tissue-specific macrophages according to their origin.

In a steady state, it is thought that AMs are derived from local proliferating precursors. Under conditions other than steady state, AMs originate from blood monocytes. Tissue-specific macrophages from brain, liver and spleen are suggested to have differentiated from local proliferating precursors [13]. A study in mice described that AMs originate from fetal blood monocytes, microglia from yolk sac macrophages and intestinal macrophages from bone marrow monocytes [21]. Identification of NRP-1 and NRP-2 expression in intestinal macrophages was not performed in this study.

In the lung, there are three main classes of macrophages, namely BMs, AMs and IMs; IVMs are also present (monocytes in the inner sides of blood capillaries). BMs are isolated from induced sputum. AMs arise from circulating blood monocytes and are found in alveolar lumens. These macrophages provide a nonspecific innate defense mechanism. As has been previously reviewed, IMs are generally considered as antigen-presenting macrophages [22]. Some studies reviewed that BMs can also defined as unattached AMs in large airways and also AMs in bronchoalveolar lavage. [23,24]. In this study, NRP-1 and NRP-2 were expressed in AMs, BMs and IVMs, but not in IMs. NRP-1 and NRP-2 expression increased in AMs under conditions of lung inflammation and lung cancer. These observations suggest that NRP-1 and NRP-2 may be related to the origin of AMs from blood monocytes (IVMs).

Generally, macrophages are divided into M1 and M2 subsets based upon their roles and profiles [25]. M1 macrophages are known to drive inflammation in response to intracellular pathogens, whereas M2 macrophages play a role in the phagocytosis of foreign pathogens and apoptotic cells [26,27]. Broadly, M1 macrophages, also called classically activated macrophages, possess pro-inflammatory properties by the secretion of interleukin-17 (IL-17), reactive oxygen intermediates, proinflammatory cytokines such as tumor necrosis factor, IL-1β, IL-8 and monocyte chemoattractive protein (MCP)-1 and mediate host defenses against a variety of bacteria, protozoa and viruses. M2 macrophages, also called alternatively activated macrophages, possess anti-inflammatory functions (mostly suppression of the inflammatory response, neutralization of the Th2 response and immunosuppression) by the secretion of IL-10, IL-12 and nitric oxide and regulate wound healing. Anti-inflammatory functions and angiogenesis near tumor islets promote tumor survival and progression [22,28].

Recently, Mox macrophages have been identified as a subset of macrophages. However, *in vitro* phagocytosis assays demonstrated that Mox macrophages are poor efferocytes and are only weakly able to phagocytose oxidized low-density lipoprotein (oxLDL), suggesting that Mox may not be able to effectively clear apoptotic cells and resolve inflammation *in vivo* [29,30]. Mixed phenotypes and a phenotypic switch in macrophage populations over time, along with an association with pathology, have been suggested in previous reviews [31,32]. As observed by IHC, M1 macrophages expressed CD68^+^, CD163^-/low^ and HO-1^-^. M2 macrophages expressed CD68^+^, CD163^high^, CD206^+^, DC-SIGN^+^ and HO-1^-^. Mox macrophages expressed CD68^+^, CD163^+^ and HO-1^+^ [32]. But in other study, HO-1 expression was investigated in M-CSF-polarized M2 macrophages otherwise HO-1 contributes to the functional polarization of M2 (MSF) macrophages [33]. In this study, NRP-1 and NRP-2 expression was observed on almost all AMs. In addition, CD68, CD163 and HO-1 expression was also observed on AMs in lung tissue adjacent to the cancer margin. Most of the macrophages subsets were positive for NRP-1 and NRP-2 expression, suggesting that NRPs expression may not correlate with macrophage differentiation.

By double IHC, the detection of 20.1% of CD68^+^CD163^-^ AMs among CD68^+^CD163^+^ AMs in lung tissue adjacent to the cancer margin suggested that AMs in lung tissue adjacent to lung cancer were mostly composed of M2 macrophages. In the tumor microenvironment, M1 macrophages can progressively differentiate to a regulatory phenotype and eventually become cells that share the characteristics of both regulatory and M2 macrophages [31]. In a previous study, increased NRP-1 and NRP-2 expression was observed during colony stimulating factor of macrophage (M-CSF)-driven differentiation of human monocytes into M2-like macrophages [34]. M2 macrophages and maturing monocytes under prolonged hypoxia can produce VEGF, which is also a ligand for NRP-1 and NRP-2, and can thus promote tumor progression and angiogenesis [35]. Since macrophages around tumor play a role in immune system for tumor progression and angiogenesis, NRPs expression on macrophages around tumor may give the pathway to tumor progression and angiogenesis. However, assessments of VEGF expression and its correlation with NRPs in AMs and blood monocytes were not performed in this study.

Because DC-SIGN is naturally expressed in human dendritic cells, its expression is also increased upon M2 macrophage differentiation of alveolar macrophages induced by M-CSF [36]. Furthermore, blood monocytes and monocyte-derived dendritic cells express NRP-1 and DC-SIGN. However, there is no study about NRP-2 expression on blood monocytes and monocyte-derived dendritic cells on pubmed-based article search. In this study, 95.8% of DC-SIGN^+^ AMs co-expressed NRP-1 and 94.9% for NRP-2, and there was a correlation between NRP^+^ AMs and DC-SIGN^+^ AMs adjacent to lung cancer. Because of the co-expression of NRPs and DC-SIGN in alveolar macrophages and the fact that NRPs is also expressed in intravascular macrophages (blood monocytes), NRPs and DC-SIGN may be suggested as their involvement in the differentiation of AMs from blood monocytes or transmigration of blood monocytes through the vascular endothelial junctions. Further investigations such as (a) factors influencing the expression of NRP-1 and NRP-2, (b) status of inflammatory response during inactivation of these factors, and (3) functional relation of these factors to DC-SIGN in M2 macrophages are suggested for future research.

## Conclusion

In this study, the expression of both NRPs, specifically in AMs among tissue-specific macrophages, was observed for the first time. This study demonstrated that NRP-1 and NRP-2 are expressed on M1, M2 and Mox macrophages. Furthermore, co-localization of NRPs and DC-SIGN in alveolar macrophages from blood monocytes was described.

## References

[1] Fujisawa H. Discovery of semaphorine receptors, neuropilin and plexin, and their functions in neural development. J Neurobiol. 2004;59:24–33. 15007824 10.1002/neu.10337

[2] Pellet-Many C, Frankel P, Jia H, Zachary I.Neuropilins: structure, function and role in disease. Biochem J. 2008;411:211–226. 10.1042/BJ20071639 18363553

[3] Raimondi C, Ruhrberg C. Neuropilin signalling in vessels, neurons and tumours. Semin Cell Dev Biol. 2013;24:172–178. 10.1016/j.semcdb.2013.01.001 23319134

[4] West DC, Rees CG, Duchesne L, Patey SJ, Terry CJ, Turnbull JE, et al Interactions of multiple heparin binding growth factors with neuropilin-1 and potentiation of the activity of fibroblast growth factor-2. J Biol Chem. 2005;280:13457–13464. 15695515 10.1074/jbc.M410924200

[5] Hsieh SH, Ying NW, Wu MH, Chiang WF, Hsu CL, Wong TY, et al Galectin-1, a novel ligand of neuropilin-1, activates VEGFR-2 signaling and modulates the migration of vascular endothelial cells. Oncogene. 2008;27:3746–3753. 10.1038/sj.onc.1211029 18223683

[6] Hu B, Guo P, Bar-Joseph I, Imanishi Y, Jarzynka MJ, Bogler O, et al Neuropilin-1 promotes human glioma progression through potentiating the activity of the HGF/SF autocrine pathway. Oncogene. 2007;26:5577–5586. 17369861 10.1038/sj.onc.1210348 PMC2846324

[7] Matsushita A, Götze T, Korc M. Hepatocyte growth factor-mediated cell invasion in pancreatic cancer cells is dependent on neuropilin-1. Cancer Res. 2007;67:10309–10316. 17974973 10.1158/0008-5472.CAN-07-3256

[8] Ball SG, Bayley C, Shuttleworth CA, Kielty CM. Neuropilin-1 regulates platelet-derived growth factor receptor signaling in mesenchymal stem cells. Biochem J. 2010;427:29–40. 10.1042/BJ20091512 20102335 PMC3441150

[9] Wild JR, Staton CA, Chapple K, Corfe BM. Neuropilins: expression and roles in the epithelium. Int J ExpPathol. 2012;93:81–103. 10.1111/j.1365-2613.2012.00810.x PMC3385701 22414290

[10] Prud'homme GJ, Glinka Y.Neuropilins are multifunctional coreceptors involved in tumor initiation, growth, metastasis and immunity. Oncotarget.2012;3:921–939. 22948112 10.18632/oncotarget.626 PMC3660061

[11] Jubb AM, Sa SM, Ratti N, Strickland LA, Schmidt M, Callahan CA, et al Neuropilin-2 expression in cancer. Histopathology. 2012;61:340–349. 10.1111/j.1365-2559.2012.04224.x 22384800

[12] Chistiakov DA, Bobryshev YV, Orekhov AN. Changes in transcriptome of macrophages in atherosclerosis. J Cell Mol Med. 2015;19:1163–1173. 10.1111/jcmm.12591 25973901 PMC4459832

[13] Landsman L, Jung S. Lung macrophages serve as obligatory intermediate between blood monocytes and alveolar macrophages. J Immunol. 2007;179:3488–3494. 17785782 10.4049/jimmunol.179.6.3488

[14] Staton CA, Kumar I, Reed MW, Brown NJ. Neuropilins in physiological and pathological angiogenesis.J Pathol. 2007;212:237–248. 17503412 10.1002/path.2182

[15] Robertson D, Savage K, Reis-Filho JS, Isacke CM. Multiple immunofluorescence labelling of formalin-fixed paraffin-embedded (FFPE) tissue. BMC Cell Biol 2008;9:13 10.1186/1471-2121-9-13 18366689 PMC2288605

[16] Kim WH, Lee SH, Jung MH, Seo JH, Kim J, Kim MA, et al Neuropilin 2 expressed in gastric cancer endothelial cells increases the proliferation and migration of endothelial cells in response to VEGF. Exp Cell Res. 2009;315:2154–2164. 10.1016/j.yexcr.2009.04.018 19409892

[17] Cai Y, Wang R, Zhao YF, Jia J, Sun ZJ, Chen XM. Expression of neuropilin-2 in salivary adenoid cystic carcinoma: Its implication in tumor progression and angiogenesis. Pathol Res Prac. 2010;206:793–799. 10.1016/j.prp.2010.08.001 20851535

[18] Bagasra O. Protocols for the *in situ* PCR-amplification and detection of mRNA and DNA sequences. Nat Protoc. 2007;2:2782–2795. 18007614 10.1038/nprot.2007.395

[19] Baltzell K, Buehring GC, Krishnamurthy S, Kuerer H, Shen HM, Sison JD. Epstein-Barr virus is seldom found in mammary epithelium of breast cancer tissue using *in situ* molecular methods. Breast Cancer Res Treat. 2012;132:267–274. 10.1007/s10549-011-1841-3 22042367

[20] Fujita H, Zhang B, Sato K, Tanaka J, Sakanaka M. Expressions of neuropilin-1, neuropilin-2 and semaphorin 3A mRNA in the rat brain after middle cerebral artery occlusion. Brain Res. 2001;914:1–14. 11578592 10.1016/s0006-8993(01)02765-2

[21] Guilliams M, De Kleer I, Henri S, Post S, Vanhoutte L, De Prijck S, et al Alveolar macrophages develop from fetal monocytes that differentiate into long-lived cells in the first week of life via GM-CSF. J Exp Med. 2013;210:1977–1992. 10.1084/jem.20131199 24043763 PMC3782041

[22] Balhara J, Gounni AS. The alveolar macrophages in asthma: a double-edged sword. Mucosal Immunol. 2012;5:605–609. 10.1038/mi.2012.74 22910216

[23] Moniuszko M, Bodzenta-Lukaszyk A, Kowal K, Dabrowska M. Bronchial macrophages in asthmatics reveal decreased CD16 expression and substantial levels of receptors for IL-10, but not IL-4 and IL-7. Folia HistochemCytobiol. 2007;45:181–189. 17951166

[24] Hussell T, Bell TJ. Alveolar macrophages: plasticity in a tissue-specific context. Nat Rev Immunol. 2014;14:81–93. 10.1038/nri3600 24445666

[25] Mantovani A, Garlanda C, Locati M. Macrophages diversity and polarization in atherosclerosis: a question of balance. Arterioscler Thromb Vasc Biol. 2009;29:1419–1423. 10.1161/ATVBAHA.108.180497 19696407

[26] Cassol E, Cassetta L, Rizzi C, Alfano M, Poli G. M1 and M2a polarization of human monocyte-derived macrophages inhibits HIV-1 replication by distinct mechanisms. J Immunol. 2009;182:6237–6246. 10.4049/jimmunol.0803447 19414777

[27] Moreira AP, Cavassani KA, Hullinger R, Rosada RS, Fong DJ, Murray L, et al Serum amyloid P attenuates M2 macrophage activation and protects against fungal spore-induced allergic airway disease. J Allergy Clin Immunol. 2010;126:712–721. 10.1016/j.jaci.2010.06.010 20673988

[28] Aber A, Alber A, Howie SE, Wallace WA, Hirani N. The role of macrophages in healing the wounded lung. Int J Exp Pathol. 2012;93:243–251. 10.1111/j.1365-2613.2012.00833.x 22774768 PMC3444980

[29] Kadl A, Meher AK, Sharma PR, Lee MY, Doran AC, Johnstone SR, et al Identification of a novel macrophages phenotype that develops in response to atherogenic phospholipids via Nrf2. Circ Res. 2010;107:737–746. 10.1161/CIRCRESAHA.109.215715 20651288 PMC2941538

[30] Wolfs IM, Donners MM, de Winther MP. Differentiation factors and cytokines in the atherosclerotic plaque micro-environment as a trigger for macrophage polarization. ThrombHaemost. 2011;106:763–771. 10.1160/TH11-05-0320 21947328

[31] Mosser DM, Edwards JP. Exploring the full spectrum of macrophage activation. Nat Rev Immunol. 2008;8:958–969. 10.1038/nri2448 19029990 PMC2724991

[32] Butcher MJ, Galkina EV. Phenotypic and functional heterogeneity of macrophages and dendritic cell subsets in the healthy and atherosclerosis-prone aorta. Front Physiol. 2012;3:44 10.3389/fphys.2012.00044 22457649 PMC3307136

[33] Sierra-Filardi E, Vega MA, Sánchez-Mateos P, Corbí AL, Puig-Kröger A, Heme Oxygenase-1 expression in M-CSF-polarized M2 macrophages contributes to LPS-induced IL-10 release. Immunobiology. 2010;215:788–95. 10.1016/j.imbio.2010.05.020 20580464

[34] Ji JD, Park-Min KH, Ivashkiv LB. Expression of function of semaphoring 3A and its receptors in human monocyte-derived macrophages. Hum Immunol. 2009;70:211–217. 19480842 10.1016/j.humimm.2009.01.026 PMC4811352

[35] Staples KJ, Sotoodehnejadnematalahi F, Pearson H, Frankenberger M, Francescut L, Ziegler-Heitbrock L, Burke B. Monocyte-derived macrophages matured under prolonged hypoxia transcriptionally up-regulate HIF-1α mRNA. Immunobiology. 2011;216:832–839. 21281980 10.1016/j.imbio.2010.12.005

[36] Hodge S, Matthews G, Mukaro V, Ahern J, Shivam A, Hodge G, et al Cigarette smoke-induced changes to alveolar macrophage phenotype and function are improved by treatment with procysteine. Am J Respir Cell Mol Biol. 2011;44:673–681. 10.1165/rcmb.2009-0459OC 20595463

